# Ambient-Stable and Durable Conductive Ag-Nanowire-Network 2-D Films Decorated with a Ti Layer

**DOI:** 10.3390/nano8050321

**Published:** 2018-05-11

**Authors:** Yoon-Mi Kim, Bu-Yeon Hwang, Ki-Wook Lee, Jin-Yeol Kim

**Affiliations:** School of Advanced Materials Engineering, Kookmin University, Seoul 136-702, Korea; yoonmi722@kookmin.ac.kr (Y.-M.K); 01087896432@kookmin.ac.kr (B.-Y.H.); kwlee7908@kookmin.ac.kr (K.-W.L.)

**Keywords:** Titanium decorated silver nanowire, conductor, decoration, ambient stable

## Abstract

Highly stable and durable conductive silver nanowire (Ag NW) network electrode films were prepared through decoration with a 5-nm-thick Ti layer. The Ag NW network 2-D films showed sheet resistance values as low as 32 ohm/sq at 88% transparency when decorated with Ti. These 2-D films exhibited a 30% increase in electrical conductivity while maintaining good stability of the films through enhanced resistance to moisture and oxygen penetration as a result of the protective effect of the Ti layer.

## 1. Introduction

Two-dimensional (2-D) conductive electrode films composed of silver nanowire (Ag NW) networks have recently attracted considerable interest as an alternative to indium tin oxide (ITO) because of their good conductance, transparency, flexibility, and mechanical ductility in addition to their ability to be prepared via a low-temperature (˂150 °C) solution process. These features make them ideal for use as transparent electrodes for flexible devices [1,2,3,4,5,6,7,8]. For such device applications, some problems related to the fabrication of Ag NW network 2-D films still need to be solved. These issues include the fact that they are easy to oxidize [9], exhibit poor thermal and chemical stabilities, and are prone to haziness due to light scattering [10] by the uneven surfaces of thick Ag NWs that result from the construction of conductor films with a junction between NW networks. However, the ambient stability of the Ag NW networks remains a major challenge because the long-term use of these electrodes is limited by their poor oxidation and thermal stabilities.

Some approaches to improving the ambient stability of Ag NW networks have been proposed, including using a functional protection layer such as ITO, TiO_2_, or ZnO deposited by atomic-layer deposition and sputtering [11,12,13]. ZnO [14,15] and TiO_2_ [16] nanoparticles have been demonstrated through a sol-gel method to form a highly efficient protecting layer over the Ag NW networks. A solution-based wet coating technique using materials such as poly(3,4-ethylenedioxythiophene): poly(styrenesulfonate) (PEDOT:PSS) [17] and reduced graphene oxide (GO) [18] has also been demonstrated to provide moisture protection for Ag NW networks. In addition, Peng et al. [18] also demonstrated that a 2-D metal oxide hybrid film composed of graphene and MnO_2_ could be integrated into a planar super-capacitor design that was even highly flexible, allowing the repeated bending and folding of the devices without significant performance loss. The 2-D film composed of titanium has been described as a thermoelectric material for energy scavenging at elevated temperatures [19] with the barrier layer properties [20]. However, recently, these metal oxide films such as SnO_2_, TiO_2_, and MnO_2_ are also widely used as a material for semiconductors, memories, super-capacitors, and energy storage elements: in particular, the application of 2-D films is becoming more prevalent in gas sensing.

**Figure 1:**
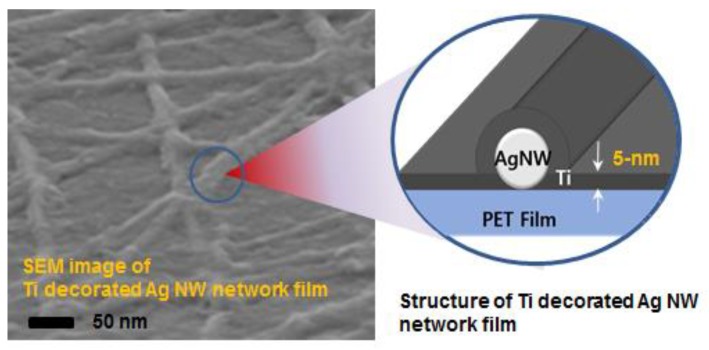
Surface structure and schematic of an Ag NW network film decorated with Ti.

In the present study, we demonstrate a highly stable and durable Ag NW network 2-D film decorated with Ti as a highly effective protecting layer, as shown in the model structure in Figure 1, for the transparent electrodes for flexible devices. Ti thin layers have good mechanical properties, including a very high specific strength and excellent corrosion resistance under extreme conditions [21,22], and they have been used as functional thin-film layers, such as in diffusion barriers, buffer layers, and inter-layers in semiconductor devices [23,24,25]. However, we have fabricated an ambient-stable and highly conductive Ag NW network electrode films by decorating the Ag NW networked 2-D film with a 5-nm-thick Ti layer using electron-beam evaporation, as shown in Figure 1. The electrical conductivity of this 2D percolating Ag NW network layer was improved by more than 30% compared with that of the as-cast Ag NW network film, while maintaining good mechanical flexibility.

## 2. Results and Discussion

Herein, Ag NWs with a diameter of 25 nm and aspect ratios as high as 1500 were directly synthesized through the modified polyol method via the via the chemical reduction of AgNO_3_ (Aldrich) in the presence of a magnetic ionic liquid, 1-butyl-3-methylimidazolium tetrachloroferrate (bmim[FeCl_4_]), using a previously reported method [26]. The synthesized Ag NWs were dispersed in an aqueous solution (0.3 wt % in H_2_O) with a polyvinylpyrrolidone (PVP) (average molecular weight, Mw = 1,300,000, Aldrich, St. Louis, MO, USA) polymer surfactant. These dispersion ink solutions are directly coated onto an O_2_-plasma-treated polyester (polyethylene terephthalate, PET) substrate film via the Meyer bar coating technique to fabricate a transparent electrode film. The density of Ag NW deposition was controlled by the volume of the coated solution, which was determined by the mesh size (#3, 5, 9, or 16) of the Mayer bar. A 3-, 5-, or 10-nm-thick-Ti layer was deposited as the decoration layer onto this 2D Ag NW network film using an electron-beam evaporator (deposition rate, 0.1 nm/s).

**Figure 2:**
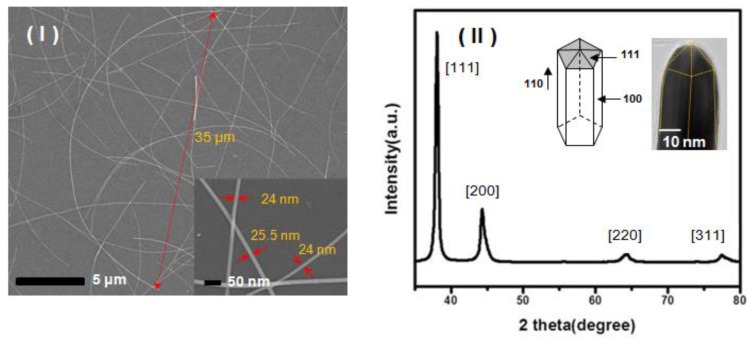
(**I**) Scanning electron microscopy (SEM) images of Ag NWs at low magnification (×2000) and high magnification (×70,000). (**II**) XRD pattern of the Ag NWs and high-resolution transmission electron microscopy (HRTEM) image of the tip of an individual pentagonal Ag NW with a diameter of 25 nm.

Scanning electron microscopy (SEM) images (Figure 2I) and a high-resolution transmission electron microscopy (HRTEM) images (Figure 2II inset) of the as-synthesized Ag NWs show that the NWs exhibit on average diameters of 25-nm with length of 35-μm capped with a 2-nm thick PVP layer. The X-ray diffraction (XRD) pattern indicates that the Ag NWs possess a face-centered cubic (fcc) crystal structure (Figure 2II). According to the previous report [27], Ag NWs synthesized were determined to have a pentagonal-twinned structure (fcc) with the surface being bound by the [100] facets and end surfaces being bound by the [111] facets. They suggested that Ag NWs grow from multiply twinned nanoparticles and that the passivation of the more active [100] surfaces by the adsorption of PVP led to the 1D growth of wires by the [111] facets. It is worth noting that the intensity ratio of the reflection at [111] and [200] exhibits relatively high values, indicating the preferred [111] orientation of the wires. The longitudinal axis was oriented along the [110] direction. In particular, as shown in the TEM image, the thickness of PVP as the surface-capping reagents of Ag NWs has been controlled at 2 nm or less in order to obtain an optimum thickness that can ensure stability at the same time while maintaining the electrical conductivity of Ag NWs.

**Figure 3:**
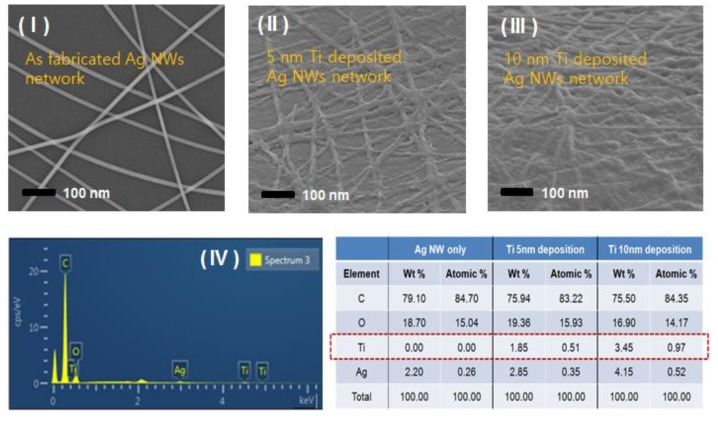
(**I**) SEM images of the as-fabricated Ag NWs network cast onto polyethylene terephthalate (PET) substrates and (**II**) 5-nm- and (**III**) 10-nm-thick-Ti deposited onto the Ag NW network. (**IV**) energy-dispersive X-ray spectroscopy (ESD) spectrum of 10-nm Ti deposited onto an Ag NW network film and the elemental analysis table for each samples.

The as-synthesized Ag NWs, after six cycles of purification by the acetone precipitation method, were suspended in DI water at an optimized density of 0.3 mg/mL and then directly coated onto an O_2_-plasma-treated PET substrate film. As a result, 2-D percolating Ag NW network layers were formed on PET films, as shown in the SEM image in Figure 3I. These 2-D network structures, which were formed when the effective network junctions were made between wires, exhibited excellent electrical characteristics. Specifically, they exhibited a low sheet resistance of 46 ohm/sq, which is a performance level that exceeds that of ITO films, even at a high transmittance of 91%.

Ti was decorated onto the Ag-NW-networked 2-D films via the electron-beam evaporation technique. Tilted-angle SEM images of a Ti-decorated Ag NW network film are presented in Figure 3II,III, which show that the Ti layer forms even 5- and 10-nm-thick coating surfaces over the entire Ag NW network, respectively. Here, the thickness of Ti layer deposited on the Ag-NW-networked 2-D layers can be controlled by the evaporation time of the electron-beam, and were measured using atomic force microscope (AFM, Nanoscope III a DI) profile of the stepped pattern formed by a difference in thickness between the Ti deposited flat surface and the uncoated substrate surface. In particular, as shown in Figure 3II, the thin 5-nm Ti fully decorated the Ag NW network; however, the thinner Ti layer with a thickness of 3 nm did not sufficiently cover the entire surface of the Ag NW network. An energy-dispersive X-ray spectroscopy (ESD) spectrum of sample 3 (Figure 3III) and the elemental analysis data for each of the above mentioned each samples are shown in Figure 3IV). In the case of samples 2 and 3, as shown in the table, the weight content of Ti relative to Ag NWs is 64.9% (weight % of Ti/Ag is 1.85/2.85) and 83.1% (weight % of Ti/Ag is 3.45/4.15) for the Ag NW networks with 5- and 10-nm-thick coatings, respectively.

**Figure 4:**
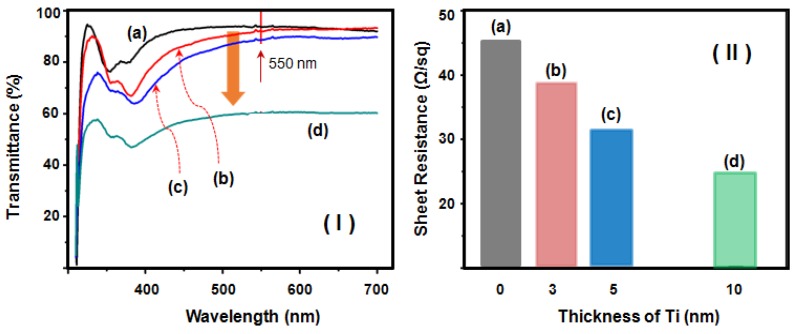
(**I**) The light transmittance spectra of (a) the as-fabricated Ag NW network cast onto a PET substrate film and of Ag NW network films with (b) 3 nm-, (c) 5 nm-, and (d) 10 nm-thick Ti. (**II**) The sheet resistances of the (a) as-fabricated Ag NW network and Ag NW network films with a (b) 3-nm-, (c) 5-nm-, or (d) 10-nm-thick Ti coating.

The sheet resistance of the 5-nm-thick-Ti-decorated Ag NW network film was 32 ohm/sq with a transmittance of 88%, which is superior to that of the undecorated Ag NW network film (46 ohm/sq with transmittance of 91%) measured using the same sample, as shown in Figure 4. However, the sheet resistance decreased substantially after deposition of the Ti layer even though the transmittance was only slightly decreased by the Ti layer. The sheet resistance was also strongly affected by the deposition thickness of the Ti layer; for example, it decreased to 25 ohm/sq when the coating thickness was increased to 10 nm. However, we speculate that this behavior is due to either the enhanced contact between the wire used for the measurement and the Ag NW network as a result of Ti deposition, induced by the shrinking force induced during the evaporation step of the deposition process; or an increase in the number of conductive pathways resulting from the conductive Ti deposited between adjacent Ag NWs.

**Figure 5:**
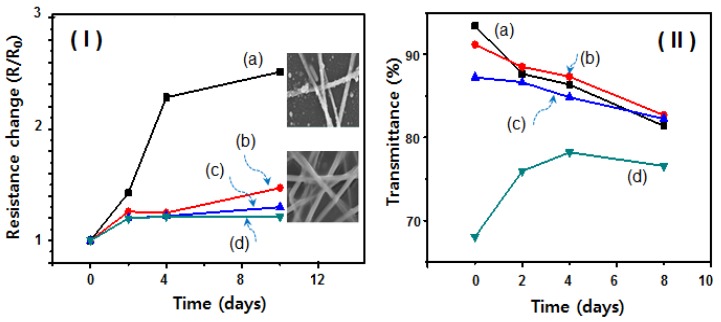
Changes in (**I**) sheet resistance and (**II**) transmittance over time under a high temperature (85 °C) and high relative humidity (85%): (a) as-fabricated Ag NW network, and Ag NW network films coated with (b) 3-nm-, (c) 5-nm-, and (d) 10-nm-thick Ti.

To further demonstrate the environmental stabilization of the conductive Ag NW network 2-D film decorated with a Ti layer, we conducted stability tests in a climate controlled chamber at 85 °C with a relative humidity of 85%. The change in the resistance value of the as-fabricated Ag NW network film after the deposition of (b) 3-, (c) 5-, and (d) 10-nm-thick Ti was measured for 10 days and the results are shown in Figure 5I. The sheet resistance of the as-fabricated Ag NW network film was fixed at approximately 46 ohm/sq, whereas extra deposition with Ti reduced the sheet resistance to approximately 32 ohm/sq (5-nm-thick Ti) and then to 25 ohm/sq (10-nm-thick Ti), as shown in Figure 4II. The Ag NW network films decorated with Ti in the 5–10 nm range exhibited only very slight changes (0.2% or less) in the relative resistance (R/R_0_) value during the stability tests, as shown in Figure 5I(c) and d. By contrast, in the case of as the fabricated Ag-NW network film, the resistance of the films increased rapidly to values at least 2.5 times greater than the relative values. In particular, the NWs within the Ag NW networks were partially oxidized, as shown in the SEM image in Figure 5I. Some of the NWs within the pristine Ag NW networks generated small droplets because of contact ripening and Rayleigh instability [28]; however, the resistance of the Ag NW layer decorated with Ti was more stable than that of the original Ag NW networks because, to some extent, the Ti layer acted as a protective layer. Figure 5II shows the change in the transmittance of the above mentioned four samples measured for 8–10 days. In the case of transmittance, the Ag NW network films decorated with Ti in the 3–5 nm thickness range were observed to gradually decrease during the stability test, as shown in Figure 5II(b,c). This phenomenon is attributable to light scattering during the partial crystallization of the Ti layer under high-temperature and high-humidity conditions. On the other hand, in the case of 10-nm-thick Ti layer (Figure 5II(d)), because the oxidation to TiO_2_ proceeds more actively than the crystallization over time, the transmittance is remarkably improved. Although Ti is a stable material in ambient conditions, it can easily react with oxygen to form a TiO_2_ coating that is visually transparent and also very stable. As a result, although the structural change to TiO_2_ partially occurred, we confirmed that the sheet resistance remained (Figure 5I(d)). However, when the conductive Ag NW network electrode film are decorated with Ti at a thickness of at least 3-nm, the stability of electric conductivity could be maintained under high-temperature and high-humidity conditions through enhanced resistance to moisture and oxygen penetration as a result of the protective effect of the Ti layer.

## 3. Conclusions

In conclusion, conductive Ag NW network electrodes decorated with a Ti layer were demonstrated to exhibit reduced sheet resistance compared with that of the undecorated Ag NW network while simultaneously exhibiting improved ambient-stability under high-temperature and high-humidity conditions. The 5-nm-thick Ti layer, despite its small thickness, exhibited excellent stability for the Ag NW network, preventing the oxidation of Ag. Additionally, the 2-D decorated Ag NW network film with a 5-nm-thick Ti layer showed a low sheet resistance of 32 ohm/sq at 88% transparency, which represents better electrical performance than ITO. Moreover, these 2-D electrode films exhibit good flexibility, making them promising candidates for use as a transparent electrode in flexible electronics. In addition, Ag NW network 2-D films decorated with Ti are very active at temperature and light and are also shown to be suitable for constructing electrode based film sensors. 2-D films of layered transition metal oxides are already commercially used in electrochemical and semiconducting gas sensing. In particular, in the case of the two-layered hybrid thin film having a high transparency and a high electric conductivity, as in the present study, it is expected that it will provide a much higher performance material in many areas of flexible transparent devices that can replace ITO.
